# Differential effects of freshwater browning across fish species: consequences for individual‐ to community‐level fish traits in north temperate lakes

**DOI:** 10.1111/brv.70074

**Published:** 2025-09-23

**Authors:** Allison M. Roth, Vincent Fugère, Marco A. Rodríguez, Jean‐François Lapierre, Joe Sánchez Schacht, Sapna Sharma, Mehdi M. Aqdam, Jeremy Fonvielle, Michelle Gros, Andrew J. Tanentzap, Matilda L. Andersson, Renee M. van Dorst, Jan Karlsson, Christopher T. Solomon, Christer Brönmark, Peter Eklöv, Kristin Scharnweber, Magnus Huss, Beatrix E. Beisner, Fernando Chaguaceda, Cristina Charette, Alison M. Derry, Gregor F. Fussmann, Andrew P. Hendry, Kaj Hulthén, Sandra Klemet‐N'Guessan, Irene Gregory‐Eaves

**Affiliations:** ^1^ University of Missouri – Columbia, Division of Biological Sciences Tucker Hall, 105, 612 Hitt St Columbia MO 65201 USA; ^2^ Department of Biology McGill University 1205 av. Dr‐Penfield, Montréal QC H3A 1B1 Canada; ^3^ Groupe de recherche interuniversitaire en limnologie (GRIL), Université de Montréal C.P. 6128, Succursale Centre‐Ville Montréal QC H3C 3J7 Canada; ^4^ Département des sciences de l'environnement Université du Québec à Trois‐Rivières 3351 Boulevard des Forges, Trois‐Rivières QC G9A 5H7 Canada; ^5^ Département de sciences biologiques, faculté des arts et sciences, Complexe des Sciences Université de Montréal 1375 Avenue Thérèse‐Lavoie‐Roux Montréal QC H2V 0B3 Canada; ^6^ Department of Biology York University 4700 Keele Street Toronto ON M3J 1P3 Canada; ^7^ Department of Biology Wilfrid Laurier University 75 University Ave W Waterloo ON N2L 3C5 Canada; ^8^ Azimuth Consulting Group Inc. 218 – 2902 West Broadway Vancouver BC V6K 2G8 Canada; ^9^ Ecosystems and Global Change Group, Department of Plant Sciences University of Cambridge Downing Street Cambridge CB2 3EA UK; ^10^ Ecosystems and Global Change Group School of the Environment, Trent University 1600 West Bank Drive Peterborough ON K9L 0G2 Canada; ^11^ Institute for Chemistry and Biology of the Marine Environment, Carl von Ossietzky University Oldenburg 26129 Germany; ^12^ Department of Aquatic Resources Swedish University of Agricultural Sciences Box 7018 Uppsala SE‐750 07 Sweden; ^13^ Department of Wildlife, Fish, and Environmental Studies Swedish University of Agricultural Sciences Skogsmarksgränd Umeå 907 36 Sweden; ^14^ Norwegian Institute for Nature Research Sognsveien 68 Oslo 0855 Norway; ^15^ Department of Ecology and Environmental Science Umeå University Linnaeus väg 6 Umeå 90187 Sweden; ^16^ Cary Institute of Ecosystem Studies Box AB Millbrook NY 12545 USA; ^17^ Functional Ecology Unit, Department of Biology Lund University Naturvetarvägen 6A Lund SE‐22362 Sweden; ^18^ Department of Ecology and Genetics/Limnology Uppsala University Norbyvägen 18 d Uppsala SE‐752 36 Sweden; ^19^ University of Potsdam, Plant Ecology and Nature Conservation Am Mühlenberg Potsdam‐Golm 314476 Germany; ^20^ University of Cologne, Ecological Research Station Rees Dores‐Albrecht‐Straße 12 Rees‐Bienen 46459 Germany; ^21^ Département des sciences biologiques Université du Québec à Montréal (UQAM) 141 Av. du Président‐Kennedy, Montréal QC H2X 1Y4 Canada; ^22^ Department of Aquatic Sciences and Assessment Swedish University of Agricultural Sciences Box 7050 Uppsala 750 07 Sweden; ^23^ Environmental and Life Sciences Graduate Program 1600 West Bank Drive, Trent University Peterborough ON K9L 0G2 Canada; ^24^ School of Environment, Resources, and Sustainability, 200 University Avenue West, University of Waterloo Waterloo ON N2L 3G1 Canada

**Keywords:** freshwater browning, fish, dissolved organic carbon, Secchi transparency, water colour, individual‐level traits, population‐level traits, community‐level traits

## Abstract

The browning of freshwater ecosystems is increasingly evident in temperate and northern regions, with widespread ramifications for lake physics, chemistry, and biology. Contrasting results on how freshwater browning may impact fish have been reported, but there has been no comprehensive examination of how browning may cause cascading effects on individual‐ to population‐ to community‐level traits of freshwater fishes. We addressed this knowledge gap by summarizing the existing literature and conducting a series of original analyses to: (*i*) explore the effects of a brown water gradient on populations of eight economically important species of fish across 871 lakes; and (*ii*) examine how a brown water gradient may influence community trait compositions across 303 lakes. From our literature synthesis, we found that fish growth is often negatively associated with browner waters, despite browning generally showing no effect on fish foraging. We also demonstrated that browner waters had greater abundances of northern pike (*Esox lucius*) and walleye (*Sander vitreus*), but lower numbers of lake trout (*Salvelinus namaycush*), yellow perch (*Perca flavescens*), largemouth bass (*Micropterus salmoides*), smallmouth bass (*M. dolomieu*), and lake whitefish (*Coregonus clupeaformis*). Moreover, we showed that fish communities were significantly more likely to contain species with larger eyes in browner lakes. Lastly, we examined relationships between various metrics of browning (i.e. dissolved organic carbon, Secchi transparency, water colour) and present a framework for how the effects of freshwater browning on fish may scale from individuals to populations to communities.

## I. INTRODUCTION

Inland waters are home to a disproportionately large diversity of fishes, hosting 40% of the world's fish species, despite representing less than 1% of all water on the planet (Dudgeon *et al*., 2006; Likens, 2009). Unfortunately, fresh waters are increasingly exposed to multiple stressors, including climate change, alterations in land use, and species invasions, all of which vary by region (Birk *et al*., 2020; Griffiths *et al*., 2022). The darkening of fresh waters – hereafter ‘browning’ – is one form of environmental change that has been altering many physical, chemical, and biological attributes of freshwater habitats over the past few decades, especially those in northeastern North America and northern Europe (Monteith *et al*., 2007; Garmo *et al*., 2014; Solomon *et al*., 2015; Meyer‐Jacob *et al*., 2019; Anderson *et al*., 2021; Räike *et al*., 2024). Analyses of water‐column time‐series data from 49 eastern Canadian lakes have revealed regionally specific trends (Imtiazy *et al*., 2025). Specifically, areas with historically intense acid deposition appear to have increased in browning from the late 1980s until ~2010, followed by a stabilizing trend or slight decline (Imtiazy *et al*., 2025). By contrast, a more remote area has demonstrated a pronounced increase in browning since ~2015 (Imtiazy *et al*., 2025).

**Fig. 1:**
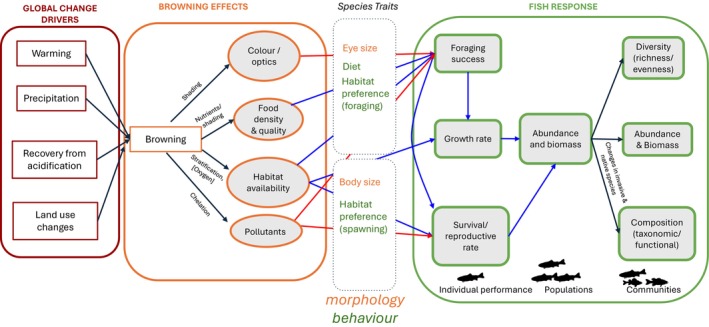
Global change drivers can trigger browning, which, in turn, influences many physical, chemical, and biological properties of inland waters. The physical, chemical, and biological changes may consequently affect organismal traits and responses in fish at the individual, population, and community levels. Blue and red arrows indicate expected increases and decreases, respectively.

Browning is most often caused by increased concentrations of terrestrial dissolved organic carbon (DOC; Monteith *et al*., 2007; Kritzberg, 2017; Anderson *et al*., 2021), although augmented iron levels may also cause browner waters (Kritzberg & Ekström, 2012; Lebret *et al*., 2018; Anderson *et al*., 2021). Anthropogenic stressors such as climate change (Weyhenmeyer & Karlsson 2009; de Wit *et al*., 2016; Meyer‐Jacob *et al*., 2019), land use change (Meyer‐Jacob *et al*., 2015; de Wit *et al*., 2016; Finstad *et al*., 2016; Kritzberg, 2017), decreased atmospheric acid deposition (Monteith *et al*., 2007; Clark *et al*., 2010; de Wit *et al*., 2021), and heightened nitrogen deposition (Rowe *et al*., 2014; Sawicka *et al*., 2017) can increase terrestrial DOC export, resulting in browning. By altering both chemical and physical properties of freshwater ecosystems (e.g. oxygen availability, light attenuation, and nutrient availability), browning may trigger ecosystem responses across spatiotemporal, biological, and ecological scales (Fig. 1; Solomon *et al*., 2015; Albrecht *et al*., 2023). For instance, Sherbo *et al*. (2023) found that browner lakes within the Experimental Lakes Area in Ontario, Canada (*N* = 286) had shallower euphotic and thermocline depths. Furthermore, within a subset of these lakes, browner lakes had lower gross and net primary productivity rates in the euphotic zone (Sherbo *et al*., 2023). Browning may also influence pelagic–benthic energy pathways (e.g. Vasconcelos *et al*., 2018, 2019; Koizumi *et al*., 2023), and there has been considerable effort dedicated to studying the effects of browning at lower trophic levels, such as plankton (reviewed in Creed *et al*., 2018; Blanchet *et al*., 2022). For example, Tanentzap *et al*. (2017) compiled stable isotope data from 147 lakes to show that the median relative contribution of terrestrially derived organic matter to zooplankton biomass was 42% and increased with DOC concentration.

Despite fish being an important structuring force in fresh waters, most current evidence detailing the effects of browning on fish is either species specific, lake specific, or experimental. As such, we do not fully understand the overarching effects of browning on fish, especially on a global scale. Furthermore, because different studies often use variable metrics (e.g. DOC concentration, Secchi transparency, water colour) to quantify browning, comparing the biological effects of browning on fish among projects can be difficult. Even a single metric like water colour may be measured in different ways (e.g. the concentration of platinum in the solution (mg Pt l^−1^), with higher concentrations indicating darker water, *versus* absorbance (*a*) at different wavelengths – often around 420 nm). For example, Robak Enbratt (2020) found that water colour (*a*_420_) was negatively correlated with both length‐at‐age for Eurasian perch (*Perca fluviatilis*) individuals at least 1 year old and length‐at‐age for individuals at least 5 years old, while total organic carbon (TOC) concentration had no effect on either response variable. These contrasts may arise because each metric reflects different aspects of browning (e.g. water colour might provide information about a fish's visual environment, whereas DOC and TOC concentration may be correlated to the amount of nutrients present for basal resources and eventually fish), and it is important to note that water colour and DOC and/or TOC are not always tightly correlated (Rodríguez‐Cardona *et al*., 2023).

Fish provide numerous fundamental ecosystem services (e.g. nutrient recycling, linking aquatic and terrestrial ecosystems, redistribution of bottom substrates) and demand‐derived services (e.g. commercial and recreational fishing, tourism; reviewed in Holmlund & Hammer, 1999; Sterner *et al*., 2020). For example, in 2018, harvests from inland fisheries represented over 12% of global fisheries production, a multibillion‐dollar industry (FAO 2020). Because environmental changes, such as increased levels of browning, may pose challenges for freshwater fish, such as decreased visibility, and ultimately may impact the vital ecosystem services fish provide, it is critical to understand the extent to which browning is influencing fish across multiple biological and spatial scales.

When examining the effects of browning on fish, we must consider that distinct levels of biological organization may respond differently (Fig. 1). In addition to contrasts among species and study systems, the strength and directionality of relationships between browning and fish traits may vary between individuals, populations, and communities. For example, browning may appear to cause beneficial increases in the biomass of a particular population, but such increases may disrupt community structure, *via* top‐down or bottom‐up effects, and alter many ecosystem functions (e.g. a decline in species diversity, nutrient recycling rates, etc.; Creed *et al*., 2018). Similarly, compensatory responses or adaptations may mask perceived responses to browning at the individual or population levels. Our lack of understanding regarding the influence of browning across multiple levels of biological organization currently limits our ability to develop and implement informed conservation and management strategies.

Here, we aim to provide a more comprehensive understanding of the effects of browning on fish by exploring the influence of browning on individual‐, population‐, and community‐level traits. We conducted a literature review detailing the findings and efforts of past researchers examining the impacts of browning on fish and paired this review with several new analyses. We quantified the effects of a brown water gradient on the abundance of eight ecologically and economically important species of fish and examined how fish community trait composition – specifically with respect to eye diameter, mouth size, and mouth position – vary along a brown water gradient. We also explored relationships among commonly reported browning metrics (i.e. DOC concentration, Secchi transparency, water colour) and discussed the implications of these relationships for understanding the effects of browning on fish. Lastly, we developed a conceptual framework for identifying how browning‐induced changes at the individual level may impact freshwater populations and, in turn, communities.

## II. METHODS

### (1) Literature review

We began our investigation into the relationships between browning and fish traits by building a metadata table to quantify the literature that has been published on this subject to date. We used this metadata table to summarize and report on the current state of the field and inform our decisions about knowledge gaps in the literature that would benefit from new analyses. We searched both *Google Scholar* and *Web of Science*, using a defined set of key words (see online Supporting Information, Table S1), between October 28, 2021 and August 20, 2022. We present the results of our search in Appendix S1. We included both peer‐reviewed articles and non‐peer‐reviewed sources (i.e. theses, non‐peer‐reviewed research reports) in our metadata table, but we omitted results relating to fish eggs or embryos. We identified 59 papers containing 305 data sets (i.e. discrete sets of browning‐related predictor variables coupled with fish‐related response variables; range of data sets per paper = 1–16; mean data sets per paper = 5.17), including both observational and experimental studies that measured the association between browning and fish traits (Appendix S1). We included studies that measured a fish response variable across either a temporal (i.e. multiple observations from the same ecosystem) or a spatial brown water gradient (i.e. observations made across different ecosystems). We did not include papers that used Secchi depth as a measure of turbidity, but we retained papers where it was clear that Secchi depth was used to measure a brown water gradient.

### (2) Tallies of past work

#### (a) Individual‐level effects of browning

Using our metadata table, we counted tallies to summarize the effects (i.e. no effect, negative linear effect, positive linear effect, negative quadratic effect, positive quadratic effect) of browning on three common response variables related to individual organisms: (*i*) fish foraging/capture rates; (*ii*) fish growth rates; and (*iii*) fish survival. For fish foraging/capture rates, we included multiple measures of fish foraging/capture rates in our tally, including number of prey captured or consumed per unit time, empty *versus* not empty stomach, ingested prey biomass, and stomach fullness. For fish growth rates, we included studies that measured growth rate as: change in mean length or mass over time, length–age data, and back‐calculated size‐at‐age from annulus widths of otoliths, cleithra, opercular bones, or other metrics. For fish survival, we included studies looking at both mortality and survival, and we switched the reported directionality of browning–mortality relationships to match browning–survival relationships. To combine studies that measured browning in different ways (e.g. DOC concentration, Secchi transparency, water colour), we considered how the response variable related to increasing water darkness, despite the directionalities of relationships reported by the authors. For example, if the authors of a study reported a negative relationship between Secchi transparency and growth rate (Devine, 2017), we added this to our tally summary as a positive relationship between browning and growth rate, given that Secchi transparency is negatively correlated with water darkness. Furthermore, because some studies examined relationships between browning and foraging/capture rates, growth rates, and/or survival in multiple species or age classes, we occasionally counted studies multiple times within each tally, with each data set within the study receiving a single count. Similarly, we included several data points per reference in our tally summary because some studies examined multiple response variables within the same category. For example, Scharnweber *et al*. (2016) measured the foraging rate of *Perca fluviatilis* on two different prey items: *Daphnia* spp. and Ephemeroptera spp. Similarly, Miller (2017) investigated two response variables related to foraging/capture rate: (*i*) proportion of zooplankton consumed in 2 h; and (*ii*) empty *versus* not empty stomachs. Similarly, some studies examined more than one browning metric. For example, Robak Enbratt (2020) examined the effect of both TOC concentration and water colour (*a*_420_) on variables related to growth rate, and we counted both relationships towards our tally results. Although this approach in counting could be viewed as non‐independent, we felt that it was better to be more inclusive, given the potential for differences in results based on how browning was measured (all metadata are available in Appendix S1). Lastly, we excluded Robbins *et al*. (2020) from our tally examining the effects of browning on growth rate, as the authors only reported results for an interaction effect, preventing us from drawing interpretations of the main effects.

### (3) Empirical data analyses

#### (a) Relationships between various browning metrics

Researchers measure the brown nature of waters using a variety of metrics (e.g. DOC concentration, Secchi transparency, water colour), yet such metrics may produce slightly different interpretations of how browning affects fish. As such, we compiled data from several large lake survey data sets from across Canada (i.e. 1533 lakes; Sandstrom, Rawson & Lester, 2010; Huot *et al*., 2019; Sánchez Schacht *et al*., 2023; Wu *et al*., 2023), the northern USA (Solomon *et al*., 2018; 127 lakes), and Europe (Miljödata‐MVM, 2023; 167 lakes) and conducted a Bayesian Principal Component Analysis (BPCA; Oba *et al*., 2003) to derive a composite measure of browning that integrated three commonly used browning metrics: DOC concentration (mg l^−1^), Secchi transparency (m), and water colour (Pt, mg l^−1^). To improve linearity and stabilize variances, we applied Box‐Cox transformations (Sakia, 1992) to DOC concentration (log), Secchi transparency (fourth‐root), and water colour (fourth‐root) prior to the BPCA. Distributional features such as kurtosis and skewness markedly differed among the original untransformed metrics; we therefore used the profile log‐likelihood generated by the *boxcox* function in the R package *MASS* (Venables & Ripley, 2002) to select an appropriate Box‐Cox transformation for each metric. BPCA has several advantages over conventional (non‐probabilistic) PCA (Oba *et al*., 2003) that were helpful in our study: (*i*) the use of a hierarchical prior provided shrinkage of component vectors such that less‐relevant principal components are automatically suppressed; (*ii*) related to the previous point, BPCA allowed us to extract only one component from the three browning metrics, in line with our goal of creating a single composite metric to measure browning; and (*iii*) the BPCA algorithm implemented an efficient expectation–maximization procedure to impute missing values for the three browning metrics, an important consideration given that, on average, 7.4% of these values were missing. The imputation procedure allowed us to include in the analysis all lakes with missing values for DOC (*N* = 187; 10.2%), Secchi (*N* = 198; 10.8%), and water colour (*N* = 23; 1.3%).

The scores from the single (first) BPCA component (BPC1; estimated using the *pcaMethods* package in R; Stacklies *et al*., 2007) accounted for 86% of the variation in the three browning metrics and were strongly correlated with the transformed DOC concentration (*r* = 0.89), Secchi transparency (*r* = −0.90), and water colour (*r* = 0.89), indicating a strong alignment between BPC1 and all three browning metrics. These correlations were stronger than those among transformed DOC, Secchi transparency, and water colour, the strongest of which (*r* = −0.70) was between DOC and Secchi transparency (Table S2). We used a graphical representation to match our BPC1 scores to the back‐transformed values of the three focal variables (i.e. DOC concentration, Secchi transparency, and water colour; Fig. S1; Appendix S2).

#### (b) Population‐level effects of browning

To assess the effects of browning on populations of eight commercially important species of fish, we obtained data on Secchi transparency (m), DOC concentration (mg l^−1^), water colour (Pt, mg l^−1^), and fish abundances (counts per lake) across 871 lakes in Ontario, Canada from the Ontario Ministry of Natural Resources Broadscale Monitoring Program. Information on lake geography (latitude, longitude), morphology (surface area, depth), and water chemistry (i.e. DOC, Secchi transparency, pH, etc.) were collected concurrently (Sandstrom *et al*., 2010; Wu *et al*., 2023).

Counts of lake trout (*Salvelinus namaycush*), northern pike (*Esox lucius*), walleye (*Sander vitreus*), yellow perch (*Perca flavescens*), largemouth bass (*Micropterus salmoides*), smallmouth bass (*Micropterus dolomieu*), lake whitefish (*Coregonus clupeaformis*), and brook trout (*Salvelinus fontinalis*) were available from between 2008 and 2017 using standardized large and small mesh gillnet surveys as part of the Ontario Ministry of Natural Resources Broad Scale Monitoring Program (Sandstrom *et al*., 2010). Large mesh gill nets were set for 16–22 h to target fish larger than 20 cm, whereas small mesh gill nets were set for 12–22 h to target fishes less than 20 cm long. Sampling effort for both types of gill net varied between lakes. For example, gill nets were set for a longer time period in larger and deeper lakes.

The fish counts are the sum across two gear types, each with their measure of nominal fishing effort. Given the synoptic nature of our analysis of browning, we focus on counts per lake aggregated over the two gears as an indicator of fish abundance, rather than examining the determinants of abundance separately for the two gears, such as in Chu *et al*. (2016). Accounting for fishing effort to achieve a consistent estimator of abundance in this context is not simple, because it is not obvious how the effort from the two gears should be combined. Additionally, effort is measured with errors arising from differences in soaking times and day of the year, which can cause errors‐in‐variables problems when adjusting for nominal effort and catchability (Cooke & Beddington, 1984; Richards & Schnute, 1986). Finally, adjusting for effort across samples is difficult even in standardized surveys, because other variables affecting catchability (e.g. fish characteristics, habitat, operating conditions) are also changing (Thompson, White & Gowan, 1998). The approach we used herein assumes that effort and catchability are unknown and must be estimated. Adjustment for these unknowns is achieved by using random effects which represent latent, lake‐specific variables that account for differences in both effort and unmeasured environmental variables that may affect catchability.

Counts of all species had a high proportion of zeros and were heavily over dispersed relative to a Poisson distribution (Fig. S2). These features of the data can lead to lack of fit, underestimation of the standard errors of estimates, and confidence intervals that are too narrow when using generalized linear mixed models (Hall, 2000; Arab *et al*., 2008; Sileshi, Hailu & Nyadzi, 2009). To account for these features of the data, we examined whether fish counts increased or declined along a brown water gradient using a zero‐inflated negative binomial (ZINB) regression model (Martin *et al*., 2005; Blasco‐Moreno *et al*., 2019; Stoklosa, Blakey & Hui, 2022). A detailed description of the model is provided in Section II.3.*c*.*i* after we highlight the main features of the model.

The counts were assumed to arise from a mixture of two distributions. The first is a Bernoulli distribution that generates ‘structural’, or ‘excess’, zeros with probability π; these zeros reflect inherent ecological restrictions that preclude a species' occurrence in a given lake, such as barriers to colonization. The second is a negative binomial distribution that generates counts, some of which may be zeros (and are usually referred to as sampling or random zeros). For the negative binomial component, we used BPC1 as the predictor, with intercept and slope varying randomly across species. We included lake identity as a random effect to account for intra‐lake correlations induced by sampling effort or environmental covariates not included in the model (e.g. maximum lake depth or surface area). The lake‐specific random effect is a proxy for effort (Thogmartin, Sauer & Knutson, 2004; Knape & Lindén, 2021) and also helps to account for the local effects of environmental variables that are not included in the model (Warton *et al*., 2015). To account for larger‐scale spatial gradients that may influence fish abundance but were not measured in this study (e.g. lake productivity, thermal regime), we also included as a predictor a smooth trend surface represented by a two‐dimensional thin‐plate spline on easting and northing lake coordinates. We modelled the mean of the negative binomial on the log scale as a linear additive function of BPC1, BPC1^2^, and the random effects. For the structural zeros component, we modelled the Bernoulli probability on the logit scale and included random effects for species and lake identity to account for variation in the probability of occurrence. We estimated model parameters in a Bayesian framework using the *brms* package in R (Bürkner, 2017, 2018). Posterior distributions were obtained from four Markov chain Monte Carlo (MCMC) chains of 7000 iterations each, with a burn‐in of 5000 iterations and a thinning factor of 2, yielding a total of 4000 retained iterations.

For each species, we used the model output to obtain the mean position of the species along a synthetic BPC1 gradient and used this position as a measure of species performance along the gradient. To calculate the mean position, we used the posterior predictive distribution of fish counts, which fully captures the uncertainty from all sources of variation to the predicted counts. First, the synthetic BPC1 gradient was represented as a set of fixed equidistant BPC1 values which covered the observed range of BPC1 values. Then, the posterior predictive distribution derived from the model was used to obtain the distribution of predicted counts at each BPC1 value. Finally, the predictive distribution of the mean position along the gradient, B, was calculated as an average of BPC1 values weighted by predicted counts:

(1) $$B_{i}=\sum_{j=1}^{M}\frac{c_{\mathrm{ij}}}{\sum_{j=1}^{M}c_{\mathrm{ij}}}x_{j},$$

where *B*_*i*_ is the predicted mean position at MCMC iteration *i* = 1, … 4000, *c*_*ij*_ is the predicted count at iteration *i* and BPC1 value *x*_*j*_ and *M* = 2001 is the number of fixed values used to represent the synthetic BPC1 gradient. The posterior predictive distribution for the mean position B was then summarized by its mean and 95% credible interval calculated across the 4000 MCMC iterations.

This analysis allowed us to explore how a composite measure of browning, which simultaneously incorporates the effects of browning on multiple key processes, such as primary production, ultraviolet (UV) protection, and optical environment for foraging may influence fish abundance. Developing a more holistic browning metric was needed as it is unclear whether DOC concentration is a consistent measure of the optical environment, given that DOC can range from highly coloured to colourless, due to differences in sources and photo‐oxidation (Massicotte *et al*., 2017). Some drivers of fish abundance, such as predator–prey interactions may instead be more strongly influenced by aspects of browning other than DOC concentration, such as water colour or transparency (Jönsson *et al*., 2013). Nevertheless, we also examined an alternative analysis by refitting the ZINB model using DOC concentration as a measure of browning instead of BPC1.

##### (i) Population model details: zero‐inflated negative binomial (ZINB) regression model with random effects

The zero‐inflated negative binomial model assumes that the excess zero counts come from a logit or probit model (and occur with probability π) and the remaining (zero or greater) counts come from a negative binomial model. The probability mass function of *y*_*ij*_, the total counts for species i=1,⋯,S=8 and lake j=1,⋯,N=871, is given by:

(2) $$p\left(y_{\mathrm{ij}}|\mu_{\mathrm{ij}},\phi \right)=\left\{\begin{array}{ll} \pi_{\mathrm{ij}}\;\text{NegBin}\left(0|\mu_{\mathrm{ij}},\phi \right), & \text{if}\;y_{\mathrm{ij}}=0\\ \left(1-\pi_{\mathrm{ij}}\right)\text{NegBin}\left(y_{\mathrm{ij}}|\mu_{\mathrm{ij}},\phi \right), & \text{if}\;y_{\mathrm{ij}}\neq 0, \end{array}\right.$$

where $\mu_{\mathrm{ij}}$ and ϕ are respectively the mean and the dispersion or scale parameters of the negative binomial distribution, and $\pi_{\mathrm{ij}}$ is the species‐ and lake‐specific probability modelled by the logit component.

The mean of the negative binomial component is modelled on the logarithmic scale with fixed and random effects as predictors:

(3) $$\log\mu_{\mathrm{ij}}=\beta_{0i}+\beta_{1i}x_{1j}+\cdots +\beta_{\mathrm{ki}}x_{\mathrm{kj}}+f\left(u_{\mathrm{kj}},v_{\mathrm{kj}}\right)+\gamma_{i}+\lambda_{j},$$

where β are regression coefficients, *x* are lake‐specific covariates (e.g. environmental measurements), *f* is a smoother term representing a two‐dimensional trend surface built on spatial coordinates *u* (easting) and *v* (northing), and γ and λ are, respectively, species‐ and lake‐specific random effects. A site‐specific random variable such as λ can be a useful proxy for ‘nuisance’ variables that impinge on abundance but are not the focus of the analysis, such as effort or detectability (Thogmartin *et al*., 2004; Knape & Lindén, 2021). It can also help account for the effects of local environmental variables that are not included in the model (Warton *et al*., 2015), whereas the spatial trend surface can represent larger‐scale non‐linear gradients (e.g. Rufener *et al*., 2017).

The logit probability of excess zeros is modelled as a function of random effects for species (η) and lake (δ):

(4) $$\text{logit}\;\pi_{\mathrm{ij}}=\eta_{i}+\delta_{j},$$

where η and δ are, respectively, species‐ and lake‐specific random effects.

##### (ii) A model for counts based on the classical catch equation

The classical catch equation assumes that catch is proportional to the product of fishing effort and density:

(5) C=qEN,

where *q* is the catchability coefficient (the fraction of the abundance that is captured by one unit of effort), *E* is the fishing effort, and *N* is a measure of fish abundance, such as the population density (Maunder & Punt, 2004).

From the catch equation it follows that the expected value of the catch for species *i* at lake *j*, $\left\langle C_{\mathrm{ij}}\right\rangle$, can be modelled on the logarithmic scale as:

(6) $$\log\left\langle C_{\mathrm{ij}}\right\rangle =\log q_{i}+\log E_{j}+\log N_{\mathrm{ij}},$$

which represents the species‐ and lake‐specific catch as a function of species‐specific catchability, lake‐specific effort, and species‐ and lake‐specific abundance.

In ecological and fisheries applications, abundance is often modelled on the logarithmic scale as a function of *k* environmental predictors and a spatial trend surface:

(7) $$\log N_{\mathrm{ij}}=\beta_{0i}+\beta_{1i}x_{1j}+\cdots +\beta_{\mathrm{ki}}x_{\mathrm{kj}}+f\left(u_{\mathrm{kj}},v_{\mathrm{kj}}\right),$$

and so:

(8) $$\log\left\langle C_{\mathrm{ij}}\right\rangle =\log q_{i}+\log E_{j}+\beta_{0i}+\beta_{1i}x_{1j}+\cdots +\beta_{\mathrm{ki}}x_{\mathrm{kj}}+f\left(u_{\mathrm{kj}},v_{\mathrm{kj}}\right).$$

If we assume that the catchability and effort terms are unknown and must be estimated, we can replace these terms with random effects:

(9) $$\log\left\langle C_{\mathrm{ij}}\right\rangle =\beta_{0i}+\beta_{1i}x_{1j}+\cdots +\beta_{\mathrm{ki}}x_{\mathrm{kj}}+f\left(u_{\mathrm{kj}},v_{\mathrm{kj}}\right)+\theta_{i}+\psi_{j},$$

where θ and ψ are, respectively, species‐ and lake‐specific random effects. Similar generalized linear mixed model formulations incorporating the catch equation are commonly used in fisheries (Candy, 2004; Maunder & Punt, 2004; Baum & Blanchard, 2010; Zhou, Campbell & Hoyle, 2019; Robertson *et al*., 2024). In these models, nominal effort is usually included as an offset or a covariate.

Equations 3 and 8 have identical structure, which shows that our approach based on ZINB regression is equivalent to a model for counts based on the classical catch equation in which effort is estimated rather than included as an offset or a covariate.

#### (c) Community‐level effects of browning

To examine how browning influences fish communities, we conducted an analysis at a larger spatial scale than the population‐level analysis and used fish occurrence (but not abundance) data that were available across Canada. We sourced complementary environmental data from the ‘Lake Pulse’ data set (Huot *et al*., 2019), a recent, standardized survey of lakes distributed across Canada. The ‘Lake Pulse’ data set provided values of transparency (m), DOC concentration (mg l^−1^), and water colour for 662 North American lakes included in the BPCA discussed above (see Section II.3.*a*). To obtain fish species checklists for all ‘Lake Pulse’ sites located in their province, we contacted provincial governments (fisheries, wildlife, or natural resource ministries). These fish data were collected through numerous standardized and non‐standardized government monitoring programs using various fishing methods; we thus treat the data as presence–absence data even when abundance information was available. Fish data were available for 332 lakes. Most sites (303 lakes) were located in the contiguous provinces of British Columbia (*N* = 159), Alberta (*N* = 28), Saskatchewan (*N* = 19), Manitoba (*N* = 4), Ontario (*N* = 19), Québec (*N* = 14), New Brunswick (*N* = 16), and Nova‐Scotia (*N* = 42). We excluded 29 lakes from other provinces or territories, as these geographically sparse data posed problems for convergence of the spatial autocorrelation term during model fitting.

We also compiled a database of 21 morphological, behavioural, and life‐history traits for all fish species in the data set. We obtained trait information from FishBase (Froese & Pauly, 2000) and Su *et al*. (2021). We used the FishBase API (Application Programming Interface) to extract data from the *species*, *morphology*, *ecology*, *food items*, *stocks*, and *maturity* tables (*rfishbase* package in R; Boettiger, Lang & Wainwright, 2012). The number of species with missing values varied per trait, with morphological traits from Su *et al*. (2021) offering the most complete trait information, including data for 100 out of 103 species. We focused the analysis on the following morphological traits for which we had *a priori* hypotheses regarding the effect of browning: (*i*) mouth size – measured as the ratio of jaw length to head diameter, which is generally larger in piscivorous fish; (*ii*) mouth position, which is the vertical position of the mouth divided by body depth and reflects feeding position in the water column (e.g. with small values (subterminal mouth) more typical of bottom feeders); and (*iii*) relative eye size – measured as the ratio of eye diameter to head diameter, with large values more typical of species with greater visual acuity (Caves, Sutton & Johnsen, 2017). We selected these traits because the effect of browning on species has been shown to vary with diet/trophic level, habitat use (benthic *versus* pelagic), and sensory ecology. More specifically, we hypothesized that browning would negatively affect benthic, piscivorous, and/or small‐eyed species. Note that all trait values correspond to measurements taken on adult specimens. To account for phylogenetic autocorrelation (closely related species responding similarly to browning, irrespective of their traits), we also obtained a phylogeny for the 100 species retained in the analysis using the R package *FishTree* (Chang *et al*., 2019).

We fitted a Bayesian joint species distribution model to the presence–absence data for each species across the 303 study lakes with the R package *Hmsc* (Tikhonov *et al*., 2022). The model had a probit error structure with BPC1 (i.e. the first BPCA component from our BPCA described in Section II.3.*a*) as a fixed effect, as well as a spatially structured random effect capturing exponential decay in correlation with increasing geodesic distance between points (using the default Gaussian process structure in *Hmsc*). The model also included the three focal traits and a phylogenetic variance–covariance matrix, obtained from the species phylogeny using the function *vcv* from the R package *ape* (assuming a Brownian motion model). Uniform priors were used for the spatial and phylogenetic terms, while default priors in *Hmsc* were used for all other model coefficients. Posterior distributions were obtained from three MCMC chains of 10,000 iterations each, with a burn‐in of 5000 iterations and a thinning factor of 5. The model was validated by inspecting MCMC traces for adequate mixing, by examining Gelman convergence diagnostic values for model coefficients and Tjur *R* ^2^ values of species‐specific models and by comparing observed *versus* predicted species prevalence and site richness. Model coefficients (effects of browning on species and traits) corresponded to the means of posterior distributions. Variance partitioning (function *computeVariancePartitioning* in *Hmsc*) was also used to assess the relative effect of browning *versus* the spatial autocorrelation on the probability of occurrence in each species‐specific model, to verify that browning was a relevant predictor of community composition.

## III. RESULTS

### (1) Individual‐level effects of browning

**Fig. 2:**
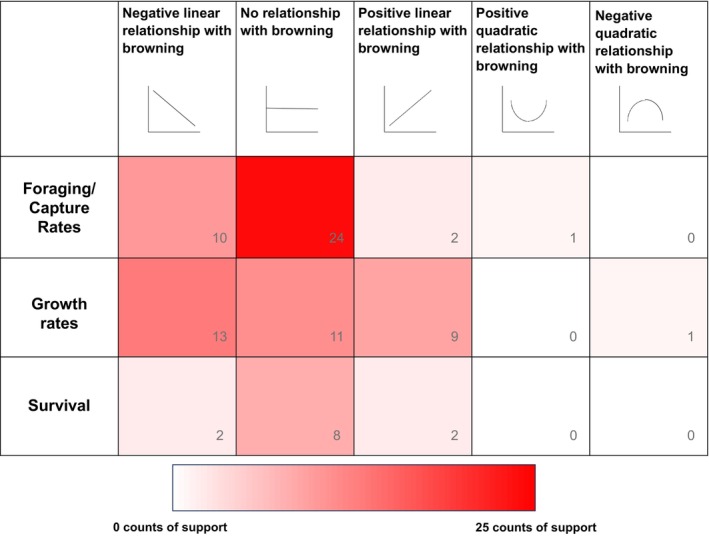
Number of data sets showing negative linear, positive linear, positive quadric, negative quadratic, or no relationship between browning and (1) foraging/capture rates, (2) growth rates, and (3) survival. Numbers in the bottom right‐hand corners indicate tally counts for each cell. See Tables S3–S5 and Appendix S1 for details.

Past studies examining the effects of browning on fish traits have primarily focused on individual‐level consequences and suggest substantial variation in the directionality of relationships between brownness and individual‐level responses, including foraging, growth, and survival (see Appendix S1). We identified 38 data sets across 20 studies that examined fish foraging/capture rates and found that there was the most evidence for no effect of browning, followed by evidence for a negative linear effect of browning, and even some evidence for a positive linear effect of browning (Fig. 2, Table S3). There was also one study that demonstrated a positive quadratic effect of browning on fish foraging/capture rates (Fig. 2, Table S3). Considering fish growth rates, we identified 35 data sets across 16 studies and found that most of the results reported a negative linear relationship between browning and fish growth rates, followed by data sets reporting no relationship between browning and fish growth rates, and data sets reporting a positive linear relationship between browning and fish growth rates (Fig. 2, Table S4). Additionally, one study suggested a negative quadratic relationship between browning and fish growth rates (Fig. 2, Table S4). Lastly, we examined 12 data sets across 10 studies for fish survival and found that most evidence suggested that there was no relationship between browning and survival, followed equally by limited evidence for both negative and positive linear effects of browning on survival (Fig. 2, Table S5).

Our compilation of the literature also revealed that there has been some research examining the effects of browning on various other facets of fish behaviour, performance, and morphology but this pool of literature is too limited to tease out trends at this point (see Appendix S1). Examples include prey selectivity (e.g. Estlander *et al*., 2010; Jönsson *et al*., 2013; Ranåker *et al*., 2014; Weidel *et al*., 2017; Leech *et al*., 2021; van Dorst *et al*., 2022), diet composition (e.g. Estlander *et al*., 2010; Bartels *et al*., 2012; Schaefer, 2014; Hedström *et al*., 2017; Koizumi *et al*., 2018; Berg, 2021), and behaviours such as attack/reaction distance, prey escape distance, encounter rate/duration, etc. (e.g. Jönsson *et al*., 2012; Ranåker *et al*., 2012, 2014; Mobley, Weigel & Boughman, 2020; see Appendix S1). There have also been several studies examining the effects of browning on non‐foraging‐related behaviours, like spawning depth (e.g. Williamson *et al*., 1997; see Appendix S1), as well as physical and performance‐related traits, such as morphology and colouration [e.g. body shape, fin shape/size, eye size, gill raker characteristics, belly/skin/fin/tail colour (e.g. Kekäläinen *et al*., 2010; Bartels *et al*., 2016; Giery & Layman, 2017; Bishop *et al*., 2022)], body condition (e.g. Hedström *et al*., 2016, 2017; Koizumi *et al*., 2018; Symons *et al*., 2019; Berg, 2021), and fecundity (e.g. Craig *et al*., 2017; see Appendix S1). These papers are generally limited in number relative to the work on foraging, growth, and survival (see Appendix S1).

### (2) Population‐level effects of browning

Our literature compilation showed that the majority of previous work examining the effects of browning on population‐level traits has focused on abundance‐related measures such as Catch Per Unit Effort (CPUE) or Biomass Per Unit Effort (BPUE), although there has been some research examining measures unrelated to abundance, such as sex ratio and size structure (e.g. Meinelt *et al*., 2004; Olin *et al*., 2017; see Appendix S1). Typically, studies have only examined one or two species.

**Fig. 3:**
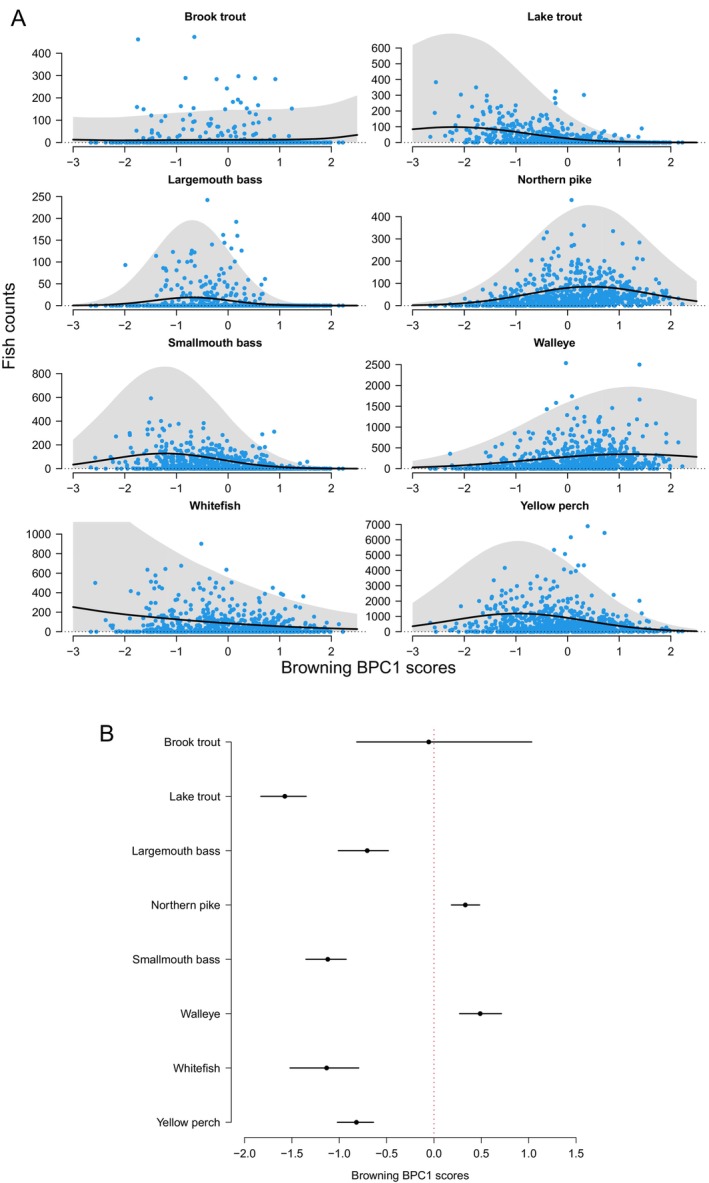
(A) Abundance (counts per lake) of eight fish species as a function of Bayesian Principal Component Analysis component 1 (BPC1; blue symbols) in 871 study lakes across Ontario, and summary for the posterior predictive distribution of the zero‐inflated negative binomial (ZINB) model (mean: black curves; 95% credible intervals: grey areas). (B) Posterior summary (mean and 95% credible interval) of the position along the BPC1 gradient.

Our analyses here expand the scope of this past work by quantitatively examining the effects of browning (measured as BPC1) on fish abundance for eight economically valuable species in Ontario lakes. Of these, we found that seven were associated with BPC1, with lower abundance in brown waters for five species (lake trout, yellow perch, largemouth bass, smallmouth bass, lake whitefish) and higher abundance in brown waters for two species (northern pike and walleye; Fig. 3). Brook trout abundance did not appear to systematically decline or increase along the brown water gradient (Fig. 3).

When we reran our analysis examining the effect of BPC1 on the abundance of these same species, using DOC concentration as the predictor rather than BPC1, our results closely mirrored those from our original analysis (Fig. S3).

### (3) Community‐level effects of browning

**Fig. 4:**
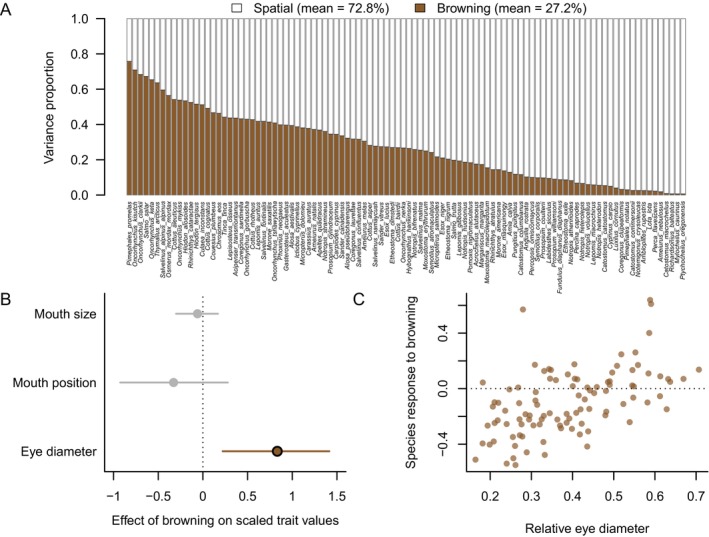
Joint species distribution model linking browning to fish communities. (A) Variance partitioning results showing the relative contribution of browning Bayesian Principal Component Analysis component 1 (PC1) *versus* the spatial (random) effect to the total variance explained by each species‐specific model (bars). Variance proportion refers to the variance in probability of occurrence across the 303 lakes; the sum for browning + the spatial term is always equal to 1. The mean proportion explained by browning *versus* space across all models is shown in the legend. (B) Response of fish traits to browning. Circles indicate model coefficients while error bars provide 95% credible intervals. Intervals overlapping zero are shown in light grey, while the one trait with strong statistical support for an effect of browning (eye diameter) is shown in brown. (C) Species response to browning (model coefficients for the browning term) *versus* species‐specific trait values for eye diameter. Each circle represents a single species. The horizontal dotted line distinguishes species that responded negatively to browning (below the line) *versus* positively to browning (above the line).

Our literature compilation identified relatively little research that examined the effects of browning at the community level, although there has been some effort to examine the association between browning metrics and community biomass, species richness, or mean community body size (see Appendix S1; e.g. Rodrigues, Fontoura & da Motta Marques, 2015; Seekell, Byström & Karlsson, 2018; Koch, 2019; Murdoch *et al*., 2021). To address this gap in the literature, we examined how lake fish communities responded to a brown to clear water gradient across Canada (Fig. S4) and linked species response to browning with three morphological traits. In a joint species distribution model including 100 species‐specific models, we found that browning contributed 27% of the variance explained by models, when averaging across all species (range = 0–78% for individual species; Fig. 4A). The remaining variance was attributable to the spatial random effect, and presumably, to other abiotic or biotic variables that are correlated in space (e.g. temperature or stocking practices). Browning lowered the probability of occurrence of most species in the data set, but species with larger eyes tended to respond more positively to browning than smaller‐eyed species, on average (Fig. 4B, C). Mouth size did not predict species responses to browning, while mouth position had a weak and uncertain effect, with subterminal mouths more typical of benthic species being loosely associated with a negative response to browning (Fig. 4B; for mouth position, the credible interval overlaps 0 but the posterior distribution is mostly negative).

## IV. DISCUSSION

Many freshwater ecosystems across northeastern North America and northern Europe are browning and experiencing physical, chemical, and biological changes related to this phenomenon (Monteith *et al*., 2007; Garmo *et al*., 2014; Meyer‐Jacob *et al*., 2019; Anderson *et al*., 2021). Our literature review on browning and fish has shown that most work has focused on individual‐level responses. Although some patterns emerged in our summary of the literature, notably that fish growth rates were often negatively correlated with browning, we found that the directionality of results was often inconsistent across studies. Herein, we explore possible reasons for the contrasting responses across studies, including the range and relative position of the gradient in browning measured, the browning metric used, and variation within and across the taxa considered. Moreover, we have developed a generalized approach to compare disparate measures of browning in the absence of locally collected data. Finally, we conducted original analyses of large fish population and community data sets and highlighted the taxa and traits that responded positively, negatively, or not at all to browning. Collectively, this body of research allowed us to build a conceptual framework to advance the field and lay the groundwork for further research.

Interestingly, we found that browning often negatively influenced fish growth rates, despite also finding that browning generally had no effect on fish foraging rates. This suggests that the indirect effects of browning on fish growth rates (i.e. the effects of browning on resource availability) may be more important than the direct effects of browning on fish growth rates (i.e. the effects of browning on foraging and capture rates). Despite some patterns emerging in our review of the literature, however, we found that often the directionality of the effects of browning on individual‐level fish traits differed across studies.

Differences between studies in the range or relative position of the browning gradient measured may produce contrasting results in the strength, directionality, or shape of relationships between browning and fish traits. For example, if one examines a small section of the browning gradient, one might find a linear relationship, whereas a quadratic/non‐linear relationship may be seen if one were to expand the range in the browning variable explored. Similarly, the change in response variables that one observes at a range which encompasses only low levels of browning may differ from what one observes at a range which encompasses only high levels of browning. Moreover, lake characteristics such as lake size can mediate the response of multiple fish attributes to browning *via* their effects on stratification, water residence time, and other properties (Kelly *et al*., 2018). We recommend that future studies examine these possibilities in more detail.

At low levels of browning, light supply is typically high, thus additions of carbon (in the forms of DOC or TOC) and the nutrients bound to it can promote primary production and increase nutrient availability to higher trophic levels (reviewed in Creed *et al*., 2018). Furthermore, DOC has also been shown to buffer the negative effects of some toxic compounds. For instance, DOC reduces the lethality of many metals (reviewed in Wood, Al‐Reasi & Smith, 2011). The composition and lability of DOC, and its relationship with mercury, can mediate methyl mercury (MeHg) bioaccumulation in fish (Braaten *et al*., 2018). At lower to intermediate DOC concentrations, DOC can enhance Hg bioaccumulation by (*i*) increasing Hg transport from land to lakes, (*ii*) stimulating microbial activity, and (*iii*) through a combination of these processes, releasing bound Hg available for subsequent methylation (Porcal *et al*., 2009; French *et al*., 2014). At higher DOC concentrations, DOC can inhibit MeHg bioaccumulation *via* formation of large, recalcitrant Hg‐DOC complexes that are less available for direct biological uptake and methylation (Barkay, Gillman & Turner, 1997; Tsui & Finlay, 2011; French *et al*., 2014). Combined, these effects could explain why positive relationships between browning and growth/survival are sometimes observed.

At high levels of browning, increased browning may decrease fish growth or survival *via* several mechanisms. For example, high DOC concentrations may depress food web productivity, including both zooplanktonic and zoobenthic prey availability for fish (Karlsson *et al*., 2009; Jones, Solomon & Weidel, 2012; Kelly *et al*., 2014; Craig *et al*., 2015; Solomon *et al*., 2015; Benoît, Beisner & Solomon, 2016; Tonin *et al*., 2022; Tang *et al*., 2023). Negative relationships between browning and zoobenthic biomass may occur as a result of browning‐mediated changes in stratification depth reducing dissolved oxygen concentrations and generating physiological restrictions on zoobenthos production (Craig *et al*., 2015; Benoît *et al*., 2016), or from browning‐mediated light attenuation reducing benthic primary production, and thus overall zoobenthos biomass (Ask *et al*., 2009; Godwin *et al*., 2014; Karlsson *et al*., 2009; Benoît *et al*., 2016). High levels of browning may also change the temperature and dissolved oxygen concentrations of aquatic systems, which may influence fish growth rates and survival directly or indirectly by imposing foraging habitat limitations (Houser, 2006; Stasko, Gunn & Johnston, 2012; Zwart *et al*., 2016; Koizumi *et al*., 2018; Moslemi‐Aqdam *et al*., 2021). Lastly, decreased visibility, arising from increased browning, may reduce or alter fish foraging rates or preferences and, therefore, affect fish growth and survival (Horppila *et al*., 2011; Stasko *et al*., 2012; Scharnweber *et al*., 2016; Hedström *et al*., 2017; Weidel *et al*., 2017; Koizumi *et al*., 2018; Leech *et al*., 2021). Thus, increasing browning may be disadvantageous for fish growth or biomass production (Finstad *et al*., 2014; Karlsson *et al*., 2015; van Dorst *et al*., 2019; Moslemi‐Aqdam *et al*., 2021), despite the fact that fish may increasingly and indirectly rely on terrestrial carbon sources.

Because studies may measure browning differently (e.g. DOC concentration, water colour, or Secchi transparency), apparent discrepancies among studies in relationships between browning and fish traits may also arise as a result of heterogeneity in the browning metrics used. For example, when local DOC concentration and water colour are weakly correlated, or when there are regional differences in relationships between DOC concentration and water colour (Lapierre *et al*., 2021; Rodríguez‐Cardona *et al*., 2023), studies examining the effects of browning on fish may arrive at different conclusions depending on the metric used. In general, we recommend that researchers measure multiple browning‐related metrics moving forward to provide a comprehensive view of how browning is operating in their system(s). In our case, we found that analyses examining the effects of browning on the abundance of eight economically important species of fish in Ontario yielded similar results, regardless of whether we used BPC1 (i.e. the first principal component of a Bayesian Principal Component Analysis which included DOC concentration, Secchi transparency, and colour) or DOC concentration as our predictor variable, providing strong support for our use of the composite metric of browning in this study. We also did not identify any strong regional signal in the relationships between BPC1 and DOC (see Fig. S5). In our systems, DOC is generally coloured and thus is a good measure of the overarching effects of browning.

It is also possible that the effect of browning may vary due to differences seen within or across taxa. Within species, variation in traits such as age, size, sex, or ecomorph (e.g. benthic *versus* limnetic) may cause individuals of the same species to respond differently to browning. For example, juveniles undergoing rapid growth may benefit more from DOC‐mediated increases in food availability compared to more slowly growing adults. Individuals from different sexes or ecomorphs may also have different energetic requirements and benefit to different degrees from DOC‐mediated increases in nutrient supply. Furthermore, males and females, or different ecomorphs, may rely on different food resources, and browning may differentially influence the availability or abundance of these disparate prey items. Similarly, inconsistencies in the effects of browning may arise because different species respond in contrasting ways to browning. For example, we might expect individuals inhabiting distinct trophic niches to respond differently to changes in browning. Indeed, in our population‐level analyses, we found that increased browning corresponded with notable declines in lake trout, yellow perch, largemouth bass, smallmouth bass, and lake whitefish, and notable increases in northern pike and walleye. Both walleye and pike tend to feed at higher trophic levels than the other species (Vander Zanden, Cabana & Rasmussen, 1997).

Interspecific differences in response to browning may also be due to variation in traits. For example, differences in eye size between species may influence how well individuals respond to browning. On one hand, species with relatively large eyes may be more robust to changes in water colour, as there may be a selective advantage of a large eye (that can house a larger pupil) which increases light‐gathering capacity, and thus sensitivity to contrast and visual acuity (Land & Nilsson, 2012; Nilsson *et al*., 2012). Relatively large eyes may thus allow for a sufficient perceptual range to maintain key activities (e.g. foraging) under darker conditions (Vinterstare *et al*., 2020). By contrast, species with larger eyes, which presumably rely mostly on vision when foraging, may show larger decreases in foraging success with increased browning, as browning should reduce visibility, compared to species with smaller eyes or species that predominantly rely on non‐visual sensory modes. Revisiting our population‐level analysis which demonstrated that, of the eight species examined, only northern pike and walleye abundance exhibited increases with browning, it is important to consider that both walleye and pike have specific adaptations that may explain their robust performance in browner waters. For example, walleye have a specialized retina that enhances low‐light visual performance (Ryder, 1977; Wahl, 1994). Similarly, pike have a well‐developed lateral line system which complements their visual abilities, and which plays an important role in prey capture (New, Fewkes & Khan, 2001). Past work has shown that pike may perform better than visually oriented prey under poor light conditions (Dobler, 1977). There could also be indirect effects on fish, whereby different taxa show varied responses to a warmer but shallower epilimnion and/or lower oxygen concentrations in the hypolimnion as browning intensifies (Stasko *et al*., 2012; Solomon *et al*., 2015). Overall, it is important to recognize that interactions between terrestrial organic matter loading and lake size could further influence species distributions along a browning gradient. For example, large deep lakes provide ideal conditions for lake trout, and such lakes are less likely to possess higher DOC concentrations (Toming *et al*., 2020). As such, it is possible that lake characteristics such as size/depth, which might be linked to DOC, could partially explain our finding that lake trout abundance decreased with increasing DOC concentrations. Indeed, we found that with the Ontario lake set, mean depth was moderately negatively correlated with the browning BPC1 scores (BPC1 ~ log(*Z*_mean_); *r* = −0.47; *N* = 867), but BPC1 scores were not significantly related to lake area measures. Whole‐lake experimental manipulations are recommended in the future to disentangle the effects of browning from differences in morphometry.

Expanding our focus to the level of freshwater fish communities, it is important to keep in mind that individual‐ and population‐level, species‐specific responses to browning can have cascading effects on community and ecosystem variables, as shown by our joint species distribution analysis that quantified the effect of browning on both community composition and fish traits. Indeed, we found that fish communities inhabiting browner lakes were significantly more likely to contain species with larger eyes, which supports our hypothesis that larger‐eyed species are better adapted to see under low‐light conditions. Overall, our results suggest that individual‐level traits have the potential to influence population abundance, which can carry over to affect community composition, highlighting the importance of studying the effects of browning across multiple biological levels.

Despite the growing body of literature examining fish responses to browning, much work is needed to gain a comprehensive understanding regarding the effects of browning on fish. We recommend that future work focuses on assessing the effects of browning on a greater range of species and traits, and we recommend increased longitudinal sampling efforts. Furthermore, studies should focus on large gradients of variables spanning both extremes of browning (i.e. from very clear to very brown water) and investigate potential non‐linear relationships between browning and fish traits. Future work should include multiple metrics related to browning (e.g. DOC concentration, Secchi transparency, water colour), rather than relying on single metrics, which may not capture all aspects of browning. Additionally, future work should consider how browning may influence the success of invasive species, and how this may, in turn, influence freshwater fish communities. Conversely, stocking could modulate relationships between browning and fish community composition by maintaining populations with maladapted traits in brown lakes, something we could not consider in our analyses given the lack of information on stocking history for the majority of our study lakes. Overall, we also need more mesocosm or whole‐lake experimental evidence to quantify population‐ and community‐level effects better, as well as ecosystem productivity changes brought about by shifts in browning. Finally, we summarized patterns in the literature but hope that with a more standardized reporting of multiple browning metrics, as well as further research, meta‐analyses can be used in the future to quantify fish responses to browning clearly.

### (1) A conceptual framework for assessing the overarching effects of browning

**Fig. 5:**
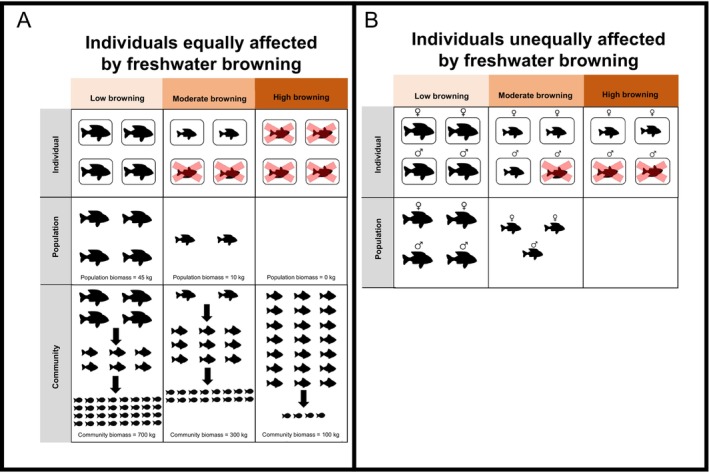
Synthesis of possible response models at different levels of browning. (A) In cases where all individuals in a population are equally affected by browning, increases in browning may reduce fish growth or survival, which may decrease population‐ and community‐level biomass (or *vice versa*). Similarly, browning‐mediated changes in growth or survival at the individual level may alter the abundance of a certain species and lead to shifts in species composition *via* (1) the removal of the species in question (direct effect) or (2) alterations in the food web structure which may trigger trophic cascades (indirect effect). (B) In cases where individuals in a population are unequally affected by browning, for example, due to different energetic requirements or prey preferences which may be mediated by browning, interindividual differences in survival may influence how populations are structured. Here, we present the example of how browning may prompt sex‐specific differences in survival, which would influence population sex ratios, but such interindividual differences could also apply to individuals from different ecomorphs or age classes.

By considering multiple levels of biological organization, we identified numerous processes that may act on fish across a browning gradient. The most widely studied aspect of browning to date is individual performance *via* behavioural or physical traits, and investigators have paid little attention to how these measurements could affect fish communities. Here we develop a couple of example scenarios to map the possible consequences of browning across multiple levels of organization (Fig. 5).

It is very plausible that the effects of browning on individual‐level performance could have population‐ and community‐level consequences. For example, browning‐related decreases in fish growth or survival may decrease population biomass (Fig. 5A). Furthermore, if different sexes, age/size classes, or ecomorphs are differentially affected by browning, this may have important consequences for the sex ratio, age/size structure, or phenotypic composition of a population (Fig. 5B). Population‐level changes in biomass may, in turn, influence community‐level biomass or composition. For example, if a given species of fish cannot survive beyond a certain browning threshold, then the loss of the focal species may cause indirect changes by altering the structure of the food web, potentially leading to trophic cascades (Fig. 5A). Our analysis of the literature, as well as our empirical analyses, highlight the complexities in combining understanding across scales, but such integration is nonetheless essential to understand the mechanistic basis for responses of fish communities to browning. Given that climatic warming can interact with browning and squeeze available habitat for coldwater fish, there is an urgent need to advance this field of science (Jane *et al*., 2024).

## V. CONCLUSIONS

(1) In this synthesis, we summarized the current literature to show that browning and fish growth rates are often negatively correlated, even though browning typically had no effect on fish foraging rates. Nevertheless, our literature review also highlighted the fact that the effects of browning on fish often vary across studies, suggesting the need for future work to expand on topics such as the range in browning gradients explored, as well as the species and trophic levels examined.
(2) We began to fill this gap in the literature by demonstrating relationships between a brown water gradient and population counts of eight economically important species of fish, where we showed that browner waters increased northern pike and walleye abundance and decreased lake trout, yellow perch, largemouth bass, smallmouth bass, and lake whitefish abundance. Although we focused on eight economically important species of fish in our analyses, future research should expand on this work and explore the effects of freshwater browning on a broader variety of species to develop a more comprehensive understanding of the effects of freshwater browning on fish populations.
(3) In addition to exploring how freshwater browning influences population‐level traits, we demonstrated that, in browner lakes, fish communities were significantly more likely to contain species with larger eyes.
(4) Lastly, we present a unique generalized approach to compare disparate measures of browning in instances where locally collected data are unavailable, providing a useful framework for future work.

## Supporting information


**Appendix S1.** Metadata table.

**Appendix S2.** Nomogram data.

**Table S1.** Defined set of key words we used in our search of the literature.
**Table S2**. Pearson pairwise correlations among browning metrics.
**Fig. S1**. Nomogram linking Bayesian Principal Component Analysis component 1 (BPC1) scores to back‐transformed values for dissolved organic carbon (DOC) concentration, Secchi transparency, and water colour.
**Fig. S2**. Distribution of catches (individual fish counts per lake) for eight species in the Ontario data set used to model the population‐level effects of browning (*N* = 871 lakes).
**Table S3**. Studies examining relationships between browning and fish foraging/capture rates.
**Table S4**. Studies examining relationships between browning and fish growth rates.
**Table S5**. Studies examining relationships between browning and fish survival (not including eggs/embryos).
**Fig. S3**. Fish abundance as a function of dissolved organic carbon (DOC) concentration, and summary for the posterior predictive distribution of the zero‐inflated negative binomial (ZINB) model; and posterior summary of the position along the log(DOC) gradient.
**Fig. S4**. Map of lakes included in the community‐level analysis, with colours representing values of browning Bayesian Principal Component Analysis component 1 (BPC1).
**Fig. S5**. Correlation between browning BPC1 scores derived from a Bayesian Principal Component Analysis (browning BPC1) and dissolved organic carbon concentrations.
