# Supplementary material for: Differential effects of freshwater browning across fish species: consequences for individual‐ to community‐level fish traits in north temperate lakes
Source: Biol Rev Camb Philos Soc. 2025 Sep 23;101(1):128–46. doi: 10.1111/brv.70074 (PMC12783421; doi:10.1111/brv.70074)
Supplement: Supplementary file 3 — Table S1. Defined set of key words we used in our search of the literature. Table S2. Pearson pairwise correlations among browning metrics. Fig. S1. Nomogram linking Bayesian Principal Component Analysis component 1 (BPC1) scores to back‐transformed values for dissolved organic carbon (DOC) concentration, Secchi transparency, and water colour. Fig. S2. Distribution of catches (individual fish counts per lake) for eight species in the Ontario data set used to model the population‐level effects of browning (N = 871 lakes). Table S3. Studies examining relationships between browning and fish foraging/capture rates. Table S4. Studies examining relationships between browning and fish growth rates. Table S5. Studies examining relationships between browning and fish survival (not including eggs/embryos). Fig. S3. Fish abundance as a function of dissolved organic carbon (DOC) concentration, and summary for the posterior predictive distribution of the zero‐inflated negative binomial (ZINB) model; and posterior summary of the position along the log(DOC) gradient. Fig. S4. Map of lakes included in the community‐level analysis, with colours representing values of browning Bayesian Principal Component Analysis component 1 (BPC1). Fig. S5. Correlation between browning BPC1 scores derived from a Bayesian Principal Component Analysis (browning BPC1) and dissolved organic carbon concentrations. [file BRV-101-128-s003.docx]

**Table S1.** Defined set of key words we used in our search of the literature.

| **Taxonomic key words** | **Browning key words** | **Response key words** |
| --- | --- | --- |
| “Fish” | “Dissolved Organic Carbon”, “DOC”, “Browning”, “Freshwater Browning”, “Lake Browning”, “River Browning”, “Brownification”, “Humic Water” | “Growth”, “Growth Rate*”, “Forag*”, “Feed*”, “Foraging Rate”, “Feeding Rate”, “Diet Composition”, “Prey Select*”, “Morpholog*”, “Surviv*”, “Mortality”, “Body Condition”, “Length”, “Recruit*”, “Fecundity”, “Product*”, “Abundance”, “CPUE”, “Biomass”, “Density”, “Sex Ratio”, “Community”, “Species Richness”, “Species Composition” |

**Table S2.** Pearson pairwise correlations among browning metrics using large lake survey data sets from across Canada (i.e. 1533 lakes; Sandstrom, Rawson & Lester, 2010; Huot *et al*., 2019; Sánchez Schacht *et al*., 2023; Wu *et al*., 2023), the northern USA (Solomon *et al*., 2018; 127 lakes), and Europe (Miljödata-MVM, 2023; 167 lakes). DOC concentration, Secchi transparency, and water colour were Box-Cox transformed (see Methods). BPC1, Bayesian Principal Components Analysis component 1; DOC, dissolved organic carbon.

|  | **DOC** | **Secchi** | **Colour** | **BPC1** |
| --- | --- | --- | --- | --- |
| **DOC** | 1.000 | –0.696 | 0.658 | 0.891 |
| **Secchi** | –0.696 | 1.000 | –0.663 | –0.898 |
| **Colour** | 0.658 | –0.663 | 1.000 | 0.886 |
| **BPC1** | 0.891 | –0.898 | 0.886 | 1.000 |

**
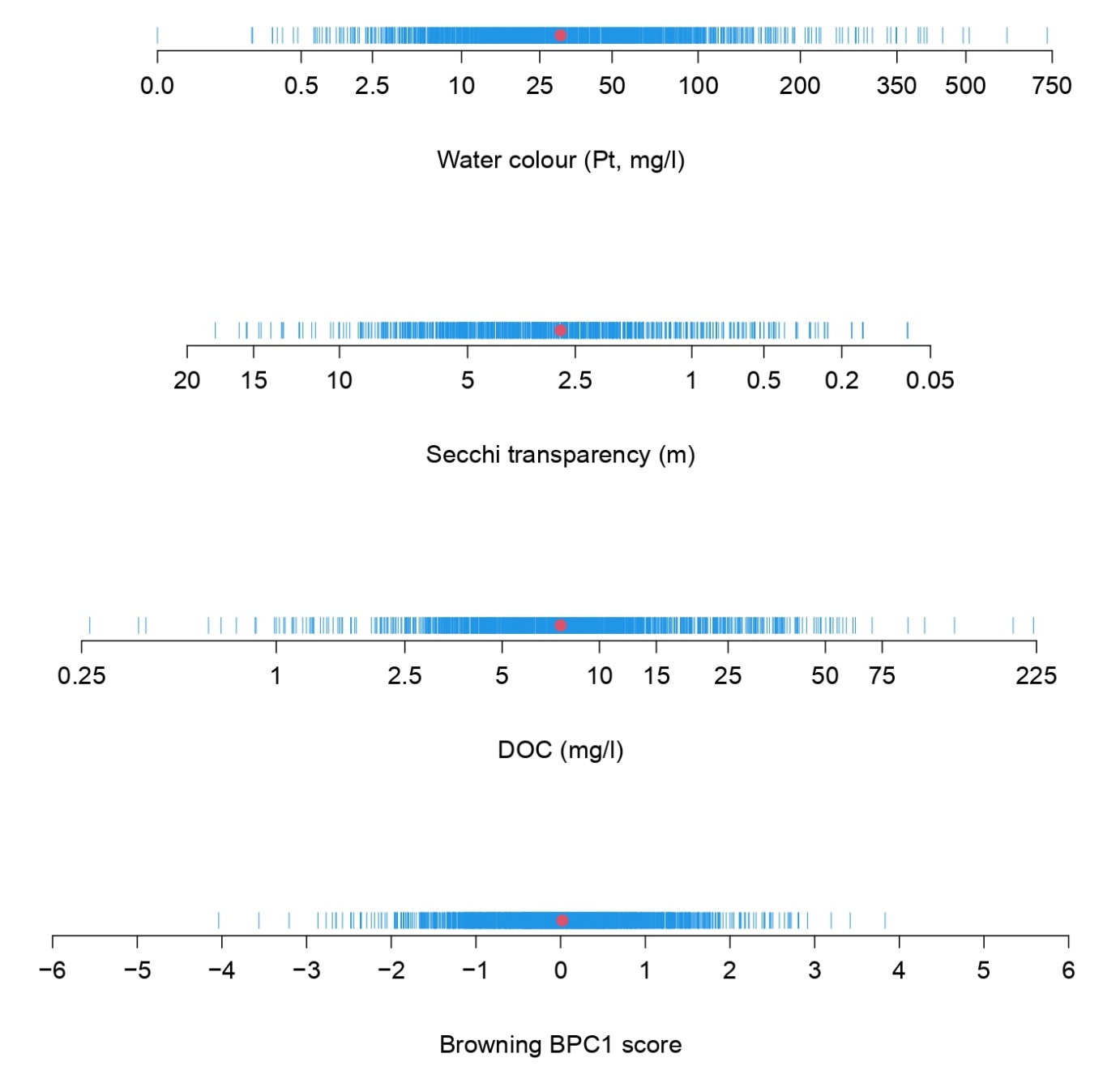
**

**Fig. S1.** Nomogram linking Bayesian Principal Components Analysis component 1 (BPC1) scores to back-transformed values for dissolved organic carbon (DOC) concentration, Secchi transparency, and water colour. For any given BPC1 score, approximate values for DOC concentration, Secchi transparency, and water colour are obtained by extending a vertical line from the BPC1 score to the corresponding scale above it. For all variables, observed values are represented as rug plots (vertical blue lines); mean values are shown as circles (red). See Appendix S2 for the data used to build this nomogram. Appendix S2 connects BPC1 to the focal variables in a one-to-one relation, both on the original (natural) scale and the transformed scale.

**
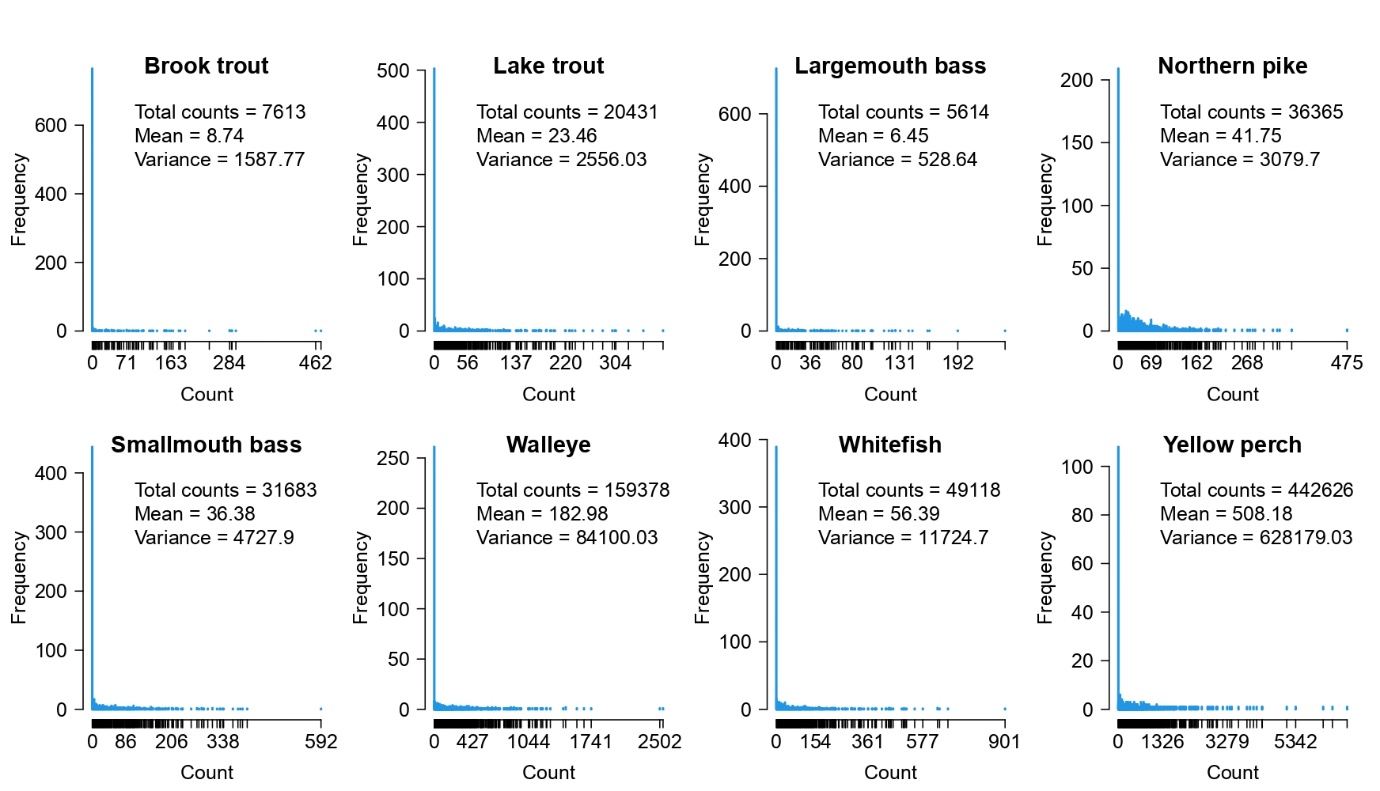
**

**Fig. S2.** Distribution of catches (individual fish counts per lake) for eight species in the Ontario data set used to model the population-level effects of browning (*N* = 871 lakes).

**Table S3.** Studies examining relationships between browning and fish foraging/capture rates. Multiple columns may contain the same study because these studies measured multiple species or ages or examined multiple response variables related to foraging/capture rates. Cells labelled 2x, 3x, etc. indicate studies that contained multiple data sets demonstrating the reported relationship.

| **Negative linear relationship with browning** | **No relationship with browning** | **Positive linear relationship with browning** | **Positive quadratic relationship with browning** |
| --- | --- | --- | --- |
| Koizumi *et al*. (2018) | Estlander *et al*. (2012) | Weidel *et al*. (2017) | Jönsson *et al* (2013) |
| Estlander *et al*. (2012) | **6x** Jönsson *et al*. (2012) | Koizumi *et al*. (2018) |  |
| Estlander *et al*. (2015) | Feuchtmayr *et al*. (2019) |  |  |
| **2x** Weidel *et al*. (2017) | **5x** Leech *et al*. (2021) |  |  |
| Hedström *et al*. (2017) | **2x** van Dorst *et al*. (2020) |  |  |
| Horppila *et al*. (2011) | **2x** Hanratty (2013) |  |  |
| Scharnweber *et al*. (2016) | Scharnweber *et al*. (2016) |  |  |
| Hanratty (2013) | Ranåker *et al*. (2014) |  |  |
| Hedström *et al*. (2016) | Lamka (2017) |  |  |
|  | **2x** Miller (2017) |  |  |
|  | Berg (2021) |  |  |
|  | Schaefer (2014) |  |  |

**Table S4.** Studies examining relationships between browning and fish growth rates. Multiple columns may contain the same study because these studies measured multiple species or ages or examined relationships with multiple metrics of browning. Cells labelled 2x, 3x, etc. indicate studies that contained multiple data sets demonstrating the reported relationship.

| **Negative linear relationship with browning** | **No relationship with browning** | **Positive linear relationship with browning** | **Negative quadratic relationship with browning** |
| --- | --- | --- | --- |
| **2x** Robak Enbratt (2020) | Leech *et al*. (2021) | Meinelt *et al*. (2004) | Giery & Layman (2017) |
| **2x** Benoît *et al*. (2016) | van Dorst *et al*. (2020) | Olin *et al*. (2017) |  |
| Symons *et al*. (2019) | Benoît *et al*. (2016) | **3x** Raitaniemi (1995) |  |
| Estlander *et al*. (2010) | **2x** Robak Enbratt (2020) | **2x** Kankaala *et al*. (2019) |  |
| **3x** van Dorst *et al*. (2019) | Estlander *et al*. (2010) | Devine (2017) |  |
| Moslemi-Aqdam *et al*. (2021) | **2x** Horppila *et al*. (2010) | van Dorst *et al*. (2020) |  |
| Kankaala *et al*. (2019) | Koizumi *et al*. (2018) |  |  |
| van Dorst *et al*. (2022) | Berg (2021) |  |  |
| Horppila *et al*. (2010) | Craig *et al*. (2017) |  |  |

**Table S5.** Studies examining relationships between browning and fish survival (not including eggs/embryos). Multiple columns may contain the same study because these studies measured multiple species or ages or examined relationships with multiple metrics of browning. Cells labelled 2x, 3x, etc. indicate studies that contained multiple data sets demonstrating the reported relationship.

| **Negative linear relationship with browning** | **No relationship with browning** | **Positive linear relationship with browning** |
| --- | --- | --- |
| Hedström *et al*. (2017) | Baldigo & Murdoch (1997) | Van Sickle *et al*. (1996) |
| Serrano *et al*. (2008) | **2x** Robak Enbratt (2020) | Simonin *et al*. (1993) |
|  | Devine (2017) |  |
|  | **2x** van Dorst *et al*. (2020) |  |
|  | Meinelt *et al*. (2004) |  |
|  | Berg (2021) |  |

**
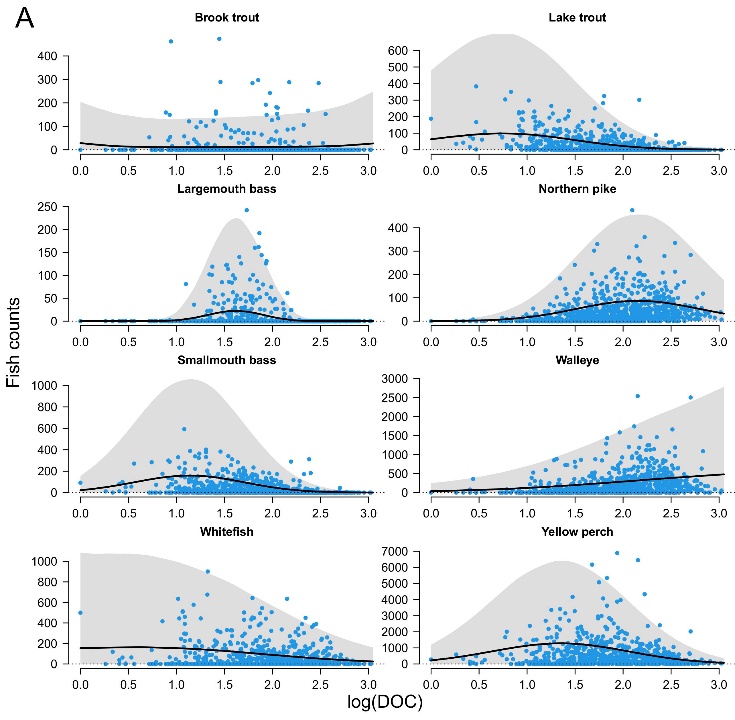
** **
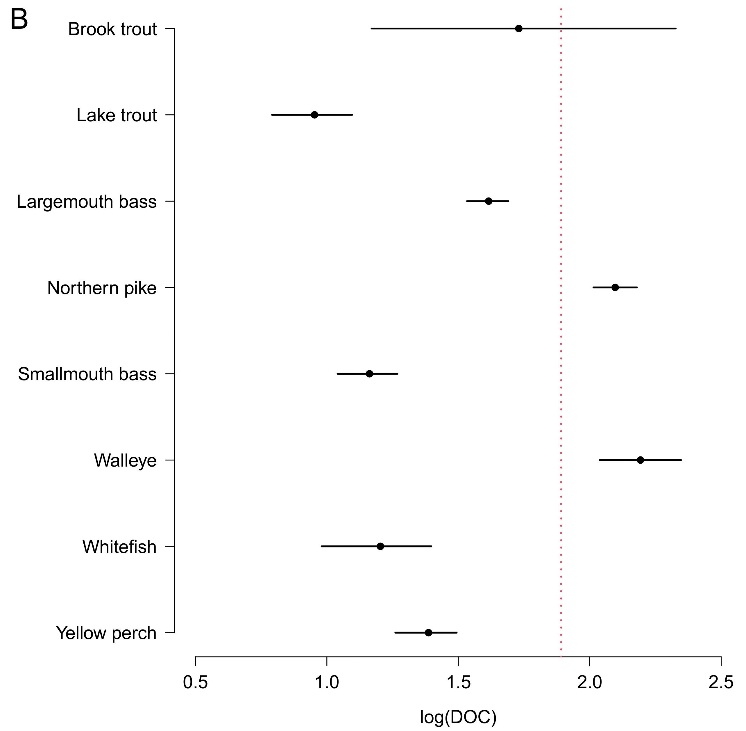
**

**Fig. S3.** (A) Fish abundance (counts: blue symbols) as a function of dissolved organic carbon (DOC) concentration (rather than Bayesian Principal Components Analysis component 1, BPC1), and summary for the posterior predictive distribution of the zero-inflated negative binomial (ZINB) model (mean: black curves; 95% credible intervals: grey areas). (B) Posterior summary (mean and 95% credible interval) of the position along the log(DOC) gradient.


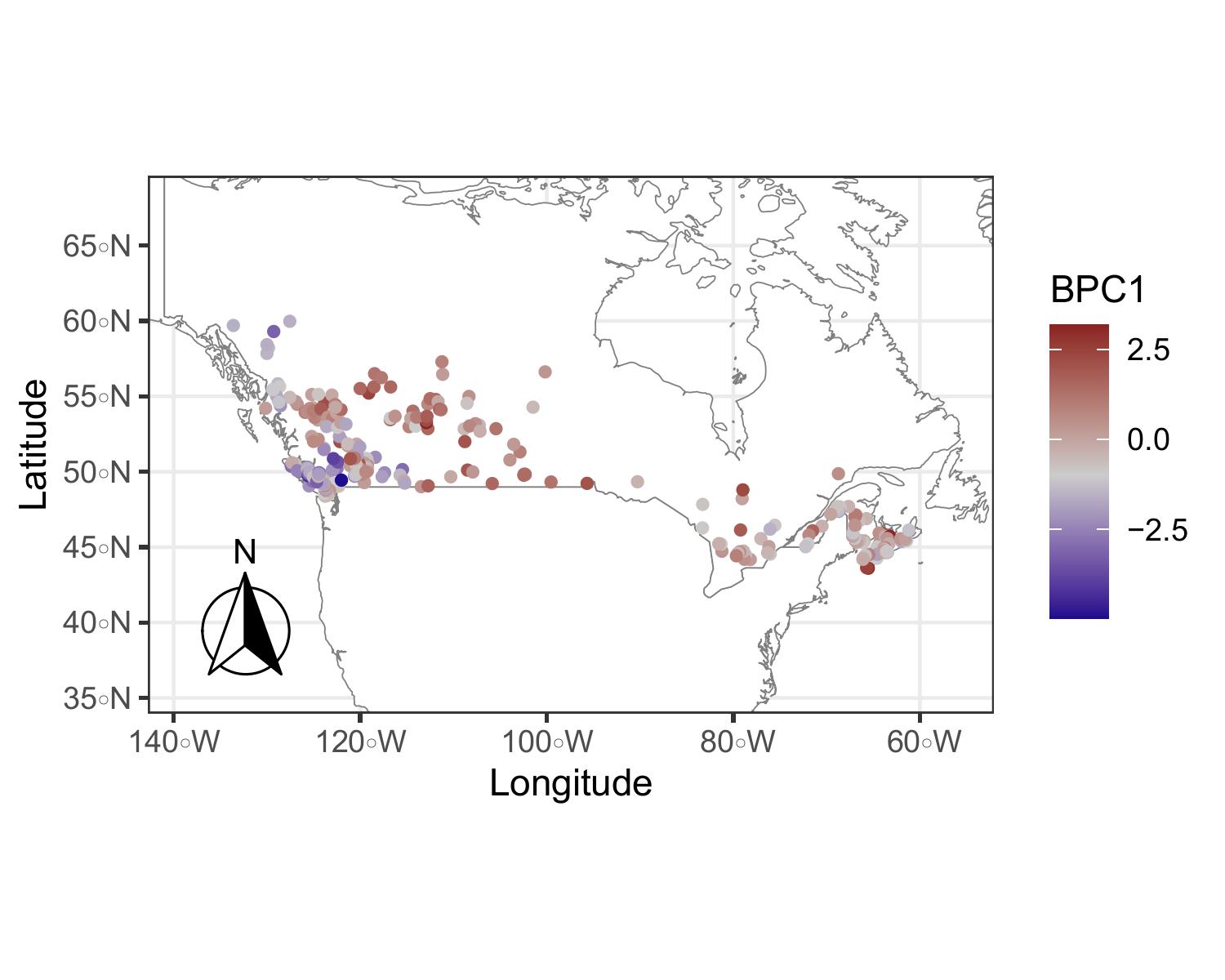


**Fig. S4.** Map of lakes included in the community-level analysis, with colours representing values of browning Bayesian Principal Components Analysis component 1 (BPC1) (negative = clear lakes, positive = darker lakes).


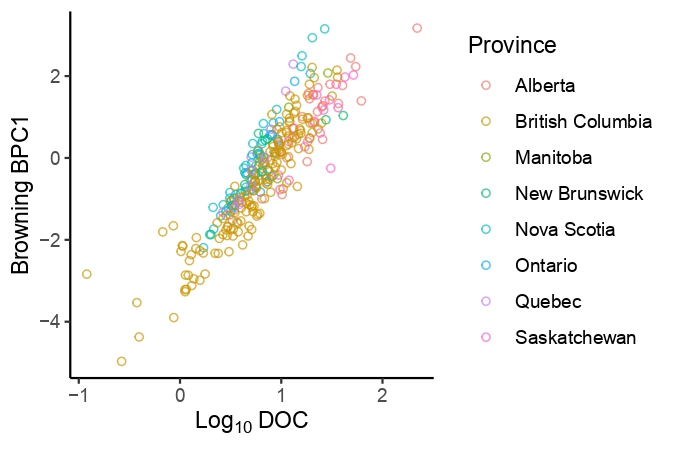


**Fig. S5.** Correlation between browning Bayesian Principal Components Analysis component 1 (BPC1) scores derived from a Bayesian Principal Component Analysis (browning BPC1) and dissolved organic carbon (DOC) concentrations.
